# (-)-Syringaresinol Exerts an Antidepressant-like Activity in Mice by Noncompetitive Inhibition of the Serotonin Transporter

**DOI:** 10.3390/ph17121637

**Published:** 2024-12-05

**Authors:** Yingyao Wu, Jianxin Cai, Hanhe Liu, Chan Li, Qingfa Tang, Yuan-Wei Zhang

**Affiliations:** 1 School of Life Sciences, Guangzhou University, Guangzhou 510006, China; 2 School of Traditional Chinese Medicine, Southern Medical University, Guangzhou 510515, China; 3 Guangdong Provincial Key Laboratory of Chinese Medicine Pharmaceutics, Guangzhou 510515, China

**Keywords:** (-)-syringaresinol, lignans, serotonin transporter, noncompetitive inhibitor, allosteric inhibition, antidepressants

## Abstract

Background: *Albizia julibrissin* Durazz. is one of the most popular herbs used for depression treatment, but the molecular basis for its mechanism of action has not been fully addressed. Previously, we isolated and identified two lignan glycoside derivatives that were shown to noncompetitively inhibit serotonin transporter (SERT) activity but with a relatively low inhibitory potency compared with those of conventional antidepressants. Methods: We characterized the pharmacological profile of the parental compound of these previously isolated lignan glycosides, (-)-syringaresinol (SYR), in inhibiting SERT by using biochemical, pharmacological, and behavioral approaches. Results: SYR, as a potent inhibitor, decreases SERT V_max_ but with little change in K_m_ for its fluorescent substrate. SYR was shown to block the conformational conversion essential for substrate transport by stabilizing SERT in an outward-open and inward-closed conformation. In addition, our molecular docking and biochemical validation demonstrated that SYR binds to an allosteric site in SERT and noncompetitively inhibits SERT transport and binding activity. Furthermore, administration of SYR was indicated to exert an antidepressant-like activity and to effectively attenuate chronic unpredictable mild stress (CUMS)-induced abnormalities in behaviors and synaptic protein expression in depressive animal models. Conclusions: This study not only provides molecular insights into the mechanism of action of *A. julibrissin* in the treatment of depression, but also opens up the possibility of development of a novel class of allosteric site-targeted therapeutic agents with an underlying mechanism of action different from that of conventional antidepressants.

## 1. Introduction

Depression is a widespread and all-age-related mental illness that has been clinically characterized by repeated depressive episodes, including anhedonia, insomnia, decreased speech, loss of interest and enjoyment, feelings of helplessness, and decreased energy [1,2]. Despite great efforts to pursue the effective treatment of depression, the current medications represented by the selective serotonin reuptake inhibitors (SSRIs) are insufficient and have many shortcomings, such as slow onset, low efficacy, and serious adverse effects [3,4,5]. Additionally, more than one-third of depressive patients are non-responsive to the SSRI antidepressants [6,7,8], supporting the development of novel therapeutic drugs with a variety of pharmacological actions that can be selected for each individual patient [9,10].

The serotonin transporter (SERT) is a polytopic membrane protein responsible for the reuptake of the neurotransmitter serotonin (5-hydroxytryptamine, 5-HT) following its release by serotonergic neurons and plays an important role in 5-HT neurotransmission [11,12]. SERT belongs to the neurotransmitter sodium symporter (NSS) family. Together with dopamine transporter (DAT) and norepinephrine transporter (NET), SERT is also a member of a subgroup named the monoamine transporters in the NSS family [13,14,15,16]. Among these transporters, SERT is of particular interest in neuropharmacology because it is the primary target for the SSRI antidepressants [17,18,19].

The structures of SERT bound with SSRIs have provided structural insights into the molecular basis for the mechanism of antidepressant inhibition of SERT activity [20,21,22]. In these high-resolution structures, SSRI molecules occupy the central binding site (S1), and thus, competitively inhibit 5-HT transport [20,21,22,23,24]. Intriguingly, the cryogenic electron microscopy (cryo-EM) structures have recently demonstrated an allosteric site (S2) that is formed in the extracellular vestibule connected to the S1 site in SERT [25]. Moreover, vilazodone has been recently revealed to bind to the S2 site in a cryo-EM structure of a vilazodone–imipramine–SERT complex [26]. This finding of an allosteric site has recently underlined the versatility of allosteric ligands and also shifted our efforts to develop novel therapeutic agents that specifically target the S2 site in SERT [26,27]. 

**Figure 1:**
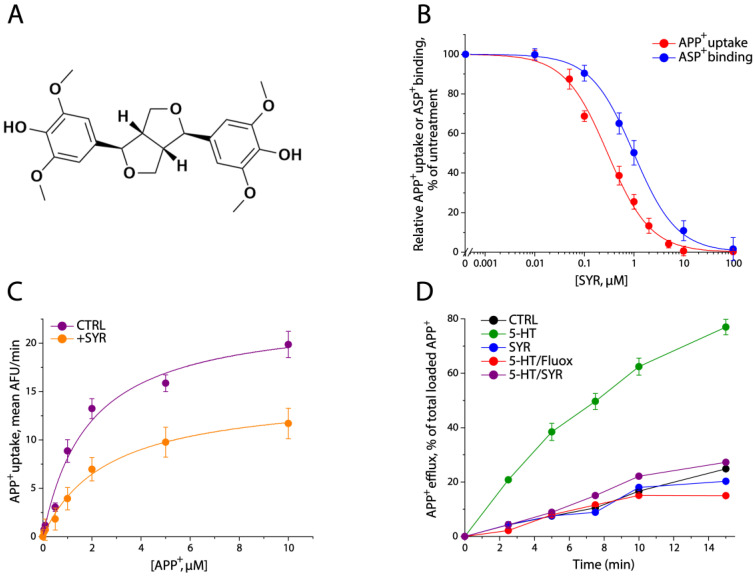
SYR noncompetitively inhibited SERT activity. (**A**) The structure of SYR. SYR holds a two-fold symmetrical structure with two 2,6-dimethoxyphenyl groups located at both sides. (**B**) SYR inhibition of both APP^+^ uptake and ASP^+^ binding by hSERT. The cells stably expressing hSERT were used for measuring APP^+^ uptake or ASP^+^ binding activity without or with SYR treatment at the indicated concentrations, as described in Section 4. The graph shows APP^+^ uptake or ASP^+^ binding activity relative to that measured in the absence of SYR. IC_50_ values for SYR inhibition of APP^+^ uptake or ASP^+^ binding were 0.25 ± 0.01 μM or 0.96 ± 0.02 μM, respectively. These IC_50_ values represent the mean ± SEM from multiple experiments (*n* ≥ 3). (**C**) Noncompetitive inhibition of APP^+^ uptake by SYR. APP^+^ uptake was measured with or without the addition of 0.25 μM SYR. K_m_ and V_max_ values represent the mean ± SEM from multiple experiments (*n* ≥ 3). (**D**) Time course of APP^+^ efflux under various treatments. The cells preloaded with APP^+^ were incubated with KRH buffer in the absence or presence of 5-HT (10 μM), SYR (10 μM), 5-HT + fluoxetine (10 μM + 10 μM), or 5-HT + SYR (10 μM + 10 μM), at 22 °C for the indicated time periods. After 3× rapid washing, the APP^+^ fluorescence retained in the cells was measured. The graph shows APP^+^ efflux expressed as a percentage of total preloaded APP^+^ fluorescence. The experiment was repeated two more times with similar results. CTRL, control; Fluox, fluoxetine.

Previously, we isolated two lignan glycoside derivatives, (-)-syringaresinol-4-O-D-apiofuranosyl-(1→2)-D-glucopyranoside (SAG) and (-)-syringaresinol-4,4′-bis-O-β-D-glucopyranoside (SBG), from *A. julibrissin,* which has been broadly used as an antidepressant drug in clinical practice. These lignan glycosides were shown to noncompetitively inhibit SERT activity [28]. Their inhibitory potency, however, was indicated to be in a few micromolar range, which is much weaker than those of the conventional SSRIs [29]. These lignan derivatives are secondary plant constituents that occur in many plants and medicinal herbs in high amounts [30,31] and share the same parental structure, (-)-syringaresinol (SYR, Figure 1A). In addition, these lignan glycosides have also been shown to be generally metabolized into SYR by gut microbes in the intestinal tract [31]. Thus, we hypothesize that the parental compound SYR may play a central structural role in the inhibition of SERT activity.

SYR is a phenolic compound that was previously shown to exert multiple pharmacological properties and potential health benefits, such as anti-inflammatory and antioxidant activities [32]; its antidepressant-like effects, however, have not been uncovered. In the present study, we characterized the natural molecule SYR in the inhibition of SERT activity and examined its antidepressant-like responses in mice by using biochemical, pharmacological, and behavioral approaches. Our results indicated that SYR, acting as an allosteric inhibitor, exerts an antidepressant-like activity through a novel underlying mechanism of action.

## 2. Results

### 2.1. SYR Noncompetitively Inhibited hSERT Activity 

SYR inhibited both 4-(4-(dimethylamino) phenyl)-1-methylpyridinium (APP^+^) uptake and 4-(4-(dimethylamino) styryl)-N-methylpyridinium (ASP^+^) binding by human SERT (hSERT). As shown in Figure 1B, APP^+^ accumulation by hSERT was inhibited by SYR, with an IC_50_ value of 0.25 ± 0.01 μM. The binding of ASP^+^ to hSERT expressed on the cell surface was also inhibited by SYR, with an IC_50_ value of 0.96 ± 0.02 μM. The inhibitory potency of SYR was increased by approximately 10-fold compared with that of its glycoside derivative, SAG [29]. The data in Figure 1C demonstrated that SYR behaved as a noncompetitive inhibitor of APP^+^ transport. The simultaneous addition of SYR with APP^+^ significantly decreased transport V_max_ but with little change in K_m_ for APP^+^. K_m_ values were 2.23 ± 0.26 μM in the control and 2.43 ± 0.20 μM in the presence of SYR, while V_max_ values were 22.98 ± 0.53 AFU/min for the control and 12.95 ± 1.52 AFU/min for the addition of 0.25 μM SYR. In separate experiments, SYR preincubation with the cells for 5 min had little effect on the kinetic parameters of inhibition. 

The data in Figure 1D demonstrated that 5-HT-stimulated APP^+^ efflux was inhibited by SYR. To exclude the possibility that SYR could also be transported by hSERT, which might cause an apparent inhibition of the fluorescent substrate APP^+^ transport, we further examined the effect of SYR on the efflux of APP^+^ by hSERT. Preloaded APP^+^ in the cells expressing hSERT was gradually released, and the basal APP^+^ efflux was remarkably stimulated by the substrate 5-HT but not by SYR. Similar to the action of a competitive inhibitor, fluoxetine, the addition of SYR effectively blocked 5-HT-induced APP^+^ efflux, supporting that SYR behaves as an inhibitor but not a substrate of hSERT. 

**Figure 2:**
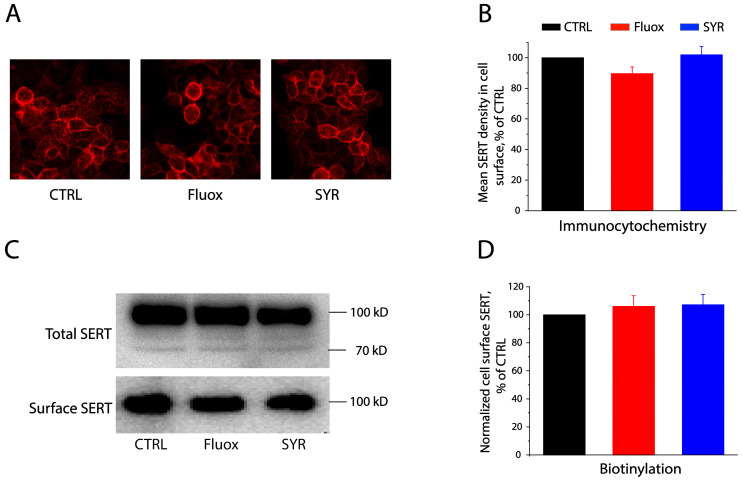
SYR effects on SERT cell surface expression. (**A**,**B**) Immunocytochemistry analysis for SERT expression on the cell surface. The cells stably expressing C-terminal Flag-tagged hSERT were treated with fluoxetine or SYR, and then incubated with anti-Flag monoclonal M2 antibody after permeabilization using 0.1% Triton X-100. The immunofluorescence images (**A**) were captured by confocal microscopy. Fluorescence intensity for SERT expression on the cell surface was quantified after subtraction of the intracellular fluorescence intensity from the total fluorescence (**B**). Error bars represent SEM from multiple experiments (*n* = 3). (**C**,**D**) Biotinylation analysis for SERT expression on the cell surface. The cells stably expressing C-terminal Flag-tagged SERT were treated with fluoxetine or SYR. SERT expressed on the cell surface was biotinylated with sulfo-NHS-SS-biotin, captured with streptavidin-agarose beads, and analyzed by immunoblot with anti-Flag monoclonal M2 antibody. Immunoreactive bands for SERT (**C**) were visualized by chemiluminescence, and quantification of SERT expression on the cell surface (**D**) was performed after normalization to total SERT expression in the cells. Error bars represent SEM from multiple experiments (*n* = 3).

To see if SYR alters hSERT cell surface expression, we conducted immunocytochemistry staining (Figure 2A,B) and biotinylation (Figure 2C,D) experiments to measure the expression level of hSERT on the cell surface with or without SYR treatments. Similar to fluoxetine, treatment with SYR at its 2× IC_50_ concentration did not alter hSERT expression on the cell surface, suggesting that SYR exerts its effects on hSERT activity by inhibiting hSERT catalytic function.

### 2.2. SYR Stabilized an Outward-Open and Inward-Closed Conformation of SERT

**Figure 3:**
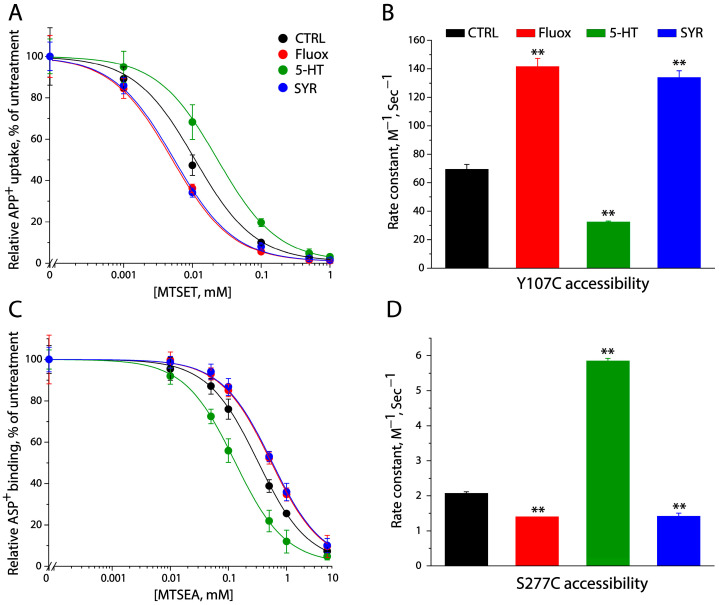
SYR stabilized an outward-open and inward-closed conformation of SERT. (**A**) MTSET concentration-dependent inhibition of APP^+^ uptake under various treatments. The inhibition of APP^+^ uptake by MTSET over a range of concentrations (0–1 mM) was measured in the cells stably expressing Y107C/C109A with or without the addition of 10 μM fluoxetine (Fluox), 5-HT, or SYR. The graph shows APP^+^ uptake relative to that in the absence of both MTSET and ligand. (**B**) Rate constants for MTSET reactivity with Y107C/C109A. Error bars represent SEM from multiple experiments (*n* ≥ 3). ** *p* < 0.01 compared with the rate constant in the control (MTSET alone). (**C**) MTSEA concentration-dependent inhibition of ASP^+^ binding under various treatments. Inhibition of ASP^+^ binding by MTSEA at the indicated concentrations was measured in digitonin-permeabilized cells stably expressing S277C/X5C with or without the addition of 10 μM fluoxetine (Fluox), 5-HT, or SYR. (**D**) Rate constants for MTSEA reactivity with S277C/X5C. Error bars represent SEM from multiple experiments (*n* ≥ 3). ** *p* < 0.01 compared with the rate constant in the control.

We used a SERT cysteine mutant, Y107C/C109A, to examine the effect of SYR on SERT conformation in the extracellular permeation pathway. Incubation with a membrane impermeant MTS reagent, 2-(trimethylammonium) ethyl methanethiosulfonate bromide (MTSET) significantly decreased Y107C/C109A activity to uptake APP^+^. The MTSET concentration-dependent inhibition was sensitive to various ligand binding events (Figure 3A). For example, fluoxetine binding led to a faster MTSET inhibition compared with the control. In contrast, incubation with substrate 5-HT together with both Na^+^ and Cl^–^ ions induced a slower inhibition. Figure 3B shows the rate constants of MTSET reactivity with Y107C/C109A. Compared with the control, fluoxetine increased the rate constant of MTSET reactivity, but the substrate decreased it, demonstrating that fluoxetine binding or substrate transport induces SERT to be in an outward-open or outward-closed conformation, respectively, consistent with our previous observation [33]. On the other hand, SYR induced the inhibition shifting to a lower MTSET concentration range (Figure 3A) and markedly increased the rate constant for MTSET reactivity with Y107C/C109A (Figure 3B), exerting a conformational effect similar to fluoxetine. 

The cysteine mutant S277C/X5C was employed as an indicator to monitor the SERT conformational state in the cytoplasmic pathway in response to SYR binding in digitonin-permeabilized cells. Treatment with 2-aminoethyl methanethiosulfonate hydrobromide (MTSEA) inhibited ASP^+^ binding in a MTSEA concentration-dependent manner (Figure 3C). Compared with the control, 5-HT transport increased the rate constant of MTSEA reactivity, indicating the substrate stabilizes an inward-open conformation in the cytoplasmic pathway, whereas either fluoxetine or SYR decreased the rate constant, representing that the binding of either fluoxetine or SYR induces an inward-closed conformation of SERT. Thus, we proposed that SYR inhibits SERT activity by stabilizing an outward-open and inward-closed conformation of SERT and consequently blocking the conformational conversion required for substrate transport.

### 2.3. Molecular Docking of SYR to the Allosteric Site in hSERT

**Figure 4:**
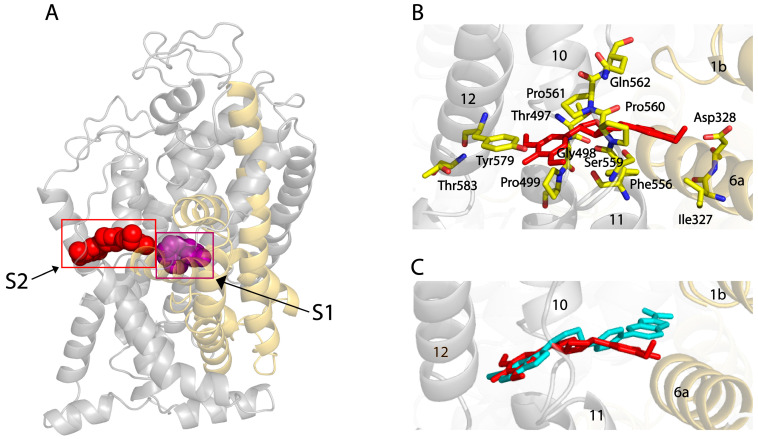
Molecular docking of SYR to the allosteric site and its binding pose in SERT. (**A**) Overall view of the hSERT–SYR–imipramine complex model in cartoon representation. SYR (red) in the allosteric binding (S2) site is depicted as spheres. Purple spheres represent the imipramine molecule bound in the central binding (S1) site. (**B**) Close-up of SYR binding in the S2 site. Residues that were proposed to interact with SYR are annotated and shown as yellow sticks. Black numbers on α-helices represent the transmembrane α-helix numbers in hSERT. (**C**) Comparison of the binding poses for vilazodone and SYR within the S2 site. The main chain positions of the bundle domain and scaffold domain of hSERT in the hSERT–imipramine–vilazodone complex structure (PDB ID, 7lWD) are shown in gold and gray, and vilazodone and SYR are shown as cyan and red sticks, respectively.

To reveal the molecular basis for SYR inhibition, we conducted molecular docking of SYR on the hSERT structure in an outward-open conformation [26]. As shown in Figure 4A,B, SYR adopts an extended conformation in an allosteric site (S2) where the SYR molecule is coordinated by the residues in several transmembrane (TM) α-helices, including Gln111 in TM1b; Ile327 and Asp328 in TM6a; Glu493, Glu494, Thr497, Gly498, and Pro499 in TM10; Phe556, Ser559, Pro560, Pro561, Gln562, Leu563, Arg564, and Leu565 in TM11; and Tyr579 and Thr583 in TM12, of which Asp328 forms an H-bond with a hydroxyl group in one 2,6-dimethoxyphenyl moiety of SYR. Another 2,6-dimethoxyphenyl moiety stretches toward TM12, interacting with the side chains of Tyr579 and Thr583. The hexahydrofurfural group is surrounded by the side chains of residues Pro499, Phe556, Pro560, and Pro561, which form an aromatic pocket in the S2 site [25]. 

Vilazodone, an allosteric inhibitor, has been recently shown to bind to the S2 site in a cryo-EM structure of the hSERT–imipramine–vilazodone complex [26]. To compare the binding poses of SYR and vilazodone in the S2 site, we superimposed SYR onto the structure of hSERT bound with vilazodone (Figure 4C). Both compounds adopt nearly linear binding poses across the S2 site. The residues binding with vilazodone or SYR are mainly from TM10, TM11, and TM12, of which several residues interacting with the moiety on one side of SYR or vilazodone toward TM12 are identical. The major difference between the vilazodone and SYR binding was also observed to involve interactions between the moiety on another side of each molecule and its interacting residues. The 2,6-dimethoxyphenyl group on another side of SYR stretches toward TM6, interacting with Asp328 by an H-bond, whereas the benzofuran carboxamide moiety of vilazodone protrudes into a subsite near the extracellular salt bridge formed by Arg104 and Glu493 [26].

### 2.4. Biochemical Validation of SYR Binding to the S2 Site in hSERT

**Figure 5:**
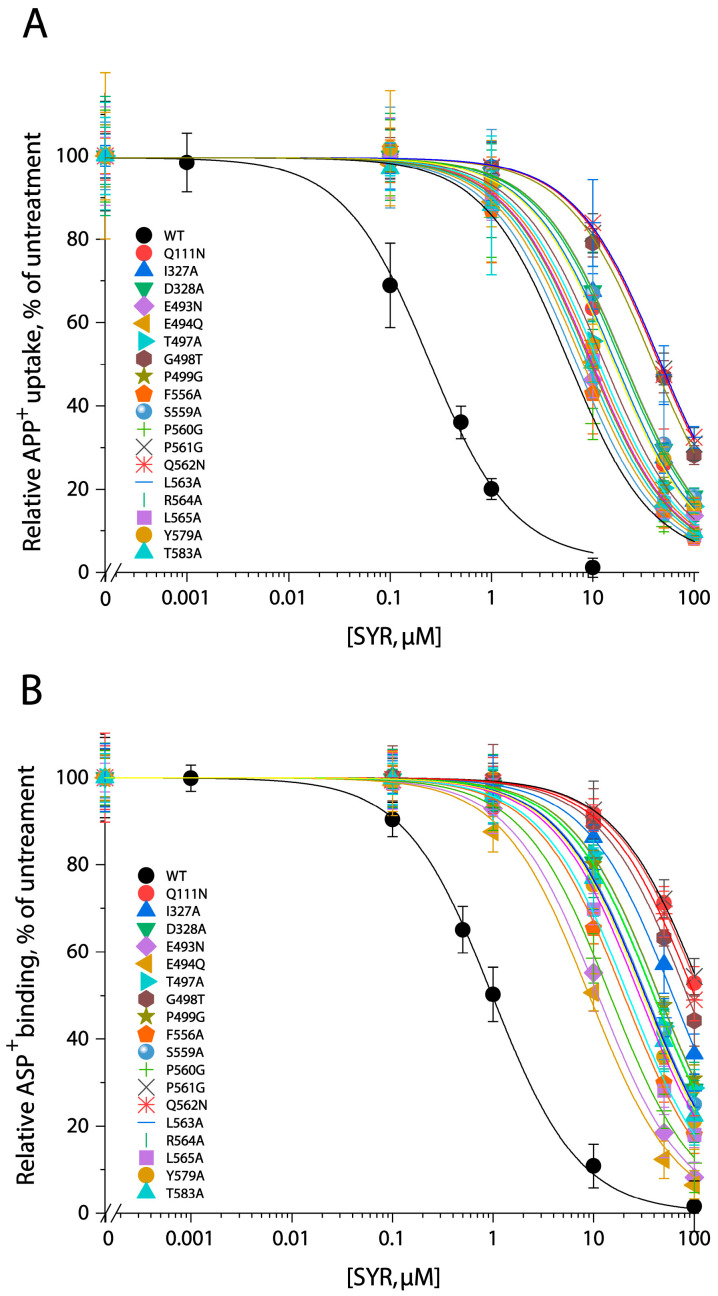
SYR inhibition of APP^+^ transport (**A**) or ASP^+^ binding (**B**) by hSERT and its S2 site mutants. APP^+^ transport or ASP^+^ binding by WT-hSERT or its allosteric S2 mutants was measured over the indicated range of SYR concentrations as described in Section 4. Nonspecific APP^+^ transport or ASP^+^ binding was measured by adding 100 μM fluoxetine. The graph shows SYR inhibition of APP^+^ transport or ASP^+^ binding by hSERT and its S2 mutants relative to that measured without SYR addition. IC_50_ values calculated from nonlinear regression analyses were shown as the mean ± SEM from at least three experiments in Table 1.

To validate SYR binding to the S2 site, we performed site-directed mutagenesis of the residues in both the allosteric and orthosteric sites and examined the influence of S1 and S2 mutations on SYR inhibitory potency against both APP^+^ transport and ASP^+^ binding by hSERT (Figure 5A,B). All S2 mutants were functional for APP^+^ uptake, with K_m_ and V_max_ values comparable to those in WT (Table 1). For 5-HT displacement, a dramatic alteration in the K_i_ value was not observed with most of the S2 mutants except for L565A, in which the K_i_ for 5-HT was significantly increased by over 100-fold compared with that in WT (Table S1), suggesting that this residue plays a critical role in 5-HT binding in the S2 site. It was also evidenced by a remarkable decrease in the K_m_ value for APP^+^ in the L565A mutant (Table 1). In comparison with WT, the IC_50_ values for either APP^+^ uptake or ASP^+^ binding in all S2 mutants tested were increased by more than 10-fold (Figure 5 and Table 1); of those, ASP^+^ binding in four mutants, Q111N, G498T, P561G, and Q562N, were inhibited by SYR with IC_50_ values approximately 100- or more fold higher than that in WT (Figure 5B and Table 1). These results demonstrated that replacement of the S2 residues in hSERT results in a dramatic reduction in SYR inhibition potency. 

By contrast, the S1 site mutations, such as Tyr95 to Phe, Ile172 to Phe, and Ser438 to Thr, which have been shown to dramatically decrease the inhibitory potency of the SSRI antidepressants such as fluoxetine and citalopram, compared with that of WT [34,35,36], had little effect on SYR inhibition of ASP^+^ binding but with a slightly decreased inhibition potency for APP^+^ uptake, with IC_50_ values 3- to 10-fold higher than that in WT (Table 1). Because these S1 mutants have been demonstrated to transport 5-HT with K_m_ values lower than that in WT [36], we speculated that the small decrease in SYR inhibition potency against APP^+^ transport is probably due to an increase in APP^+^ binding affinity caused by the mutations rather than SYR binding in the S2 site. Therefore, our results suggest that mutations of the S1 site do not interfere with SYR binding.

### 2.5. The Antidepressant-like Effects of SYR on Animal Behaviors

**Figure 6:**
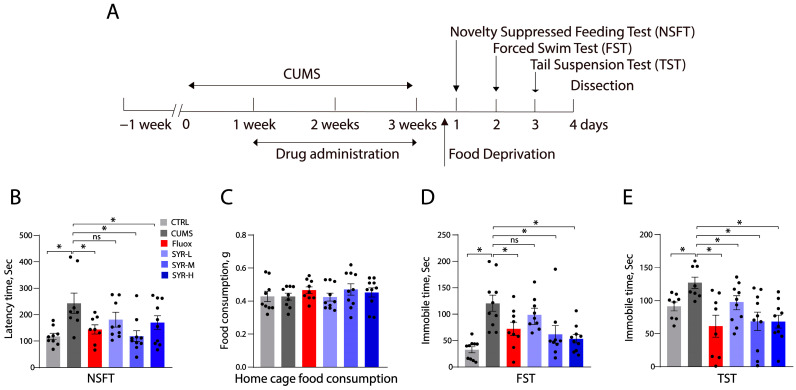
Animal behavioral tests. (**A**) Schematic illustration of animal behavioral test timeline. Mice were exposed to CUMS for 3 weeks, as described in Section 4. Fluoxetine (10 mg/kg) or SYR (5, 10, or 20 mg/kg) was administered (i.p.) once per day from day 7 to day 21. For the CUMS group, saline was given instead of drugs. NSFT was performed after 16–18 h food deprivation, followed by FST and TST. (**B**) NSFT. The latency time to food in NSFT was counted. (**C**) Home cage food consumption. Food consumed by individual mice in the home cage (g) within a 20 min period was measured after NSFT. (**D**) FST. The immobility time during a 2 to 6 min period in FST was measured. (**E**) TST. The immobility time during 2 to 6 min was counted. *n* = 8–10 mice/group. Bars represent the mean ± SEM. * *p* < 0.05 compared with the control or CUMS group, respectively, by one-way ANOVA followed by post hoc tests. ns, not significant.

To learn if SYR exhibits antidepressant-like activity in mice, we conducted several behavioral tests with chronic unpredictable mild stress (CUMS)-induced mouse models under various drug treatments (Figure 6A). Consistent with previous observations [37], the CUMS-induced increase in the time for latency to food in the novelty suppressed feeding test (NSFT) (Figure 6B) and immobility in the forced swim test (FST) (Figure 6D) or tail suspension test (TST) (Figure 6E) were remarkably alleviated by administration of fluoxetine (10 mg/kg, i.p.) for 2 weeks. By comparison, CUMS mice administered with SYR at a lower dosage (5 mg/kg, i.p.) did not exhibit a significant response to reverse behavioral abnormalities in both NSFT and FST, although the same treatment was showed to effectively attenuate the CUMS-induced increase in immobility time in TST. However, increased dosages of SYR to 10 or 20 mg/kg (i.p.) produced remarkable antidepressant-like effects on all behavioral tests. Additionally, it should be pointed out that administration of either fluoxetine or SYR at all tested dosages had little effect on home cage food consumption (Figure 6B) and daily body weight changes (Figure S1) in comparison with the control group and that administered mice kept a zero fatality rate, indicating that SYR did not display detectable toxic or adverse effects in our animal models. These behavioral observations demonstrated that SYR exerts an antidepressant-like activity comparable to the conventional SSRI inhibitor fluoxetine in CUMS mouse models.

### 2.6. The Effects of SYR on Neuronal Activity and Synaptic Proteins in mPFC and Hippocampus of Mouse Models

**Figure 7:**
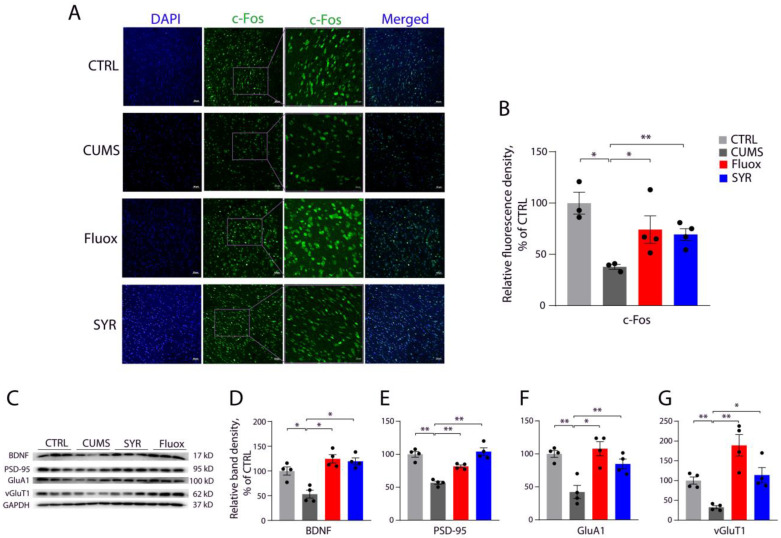
SYR effects on the expression of c-Fos and synaptic proteins in mPFC. (**A**) Representative immunohistochemical images of c-Fos expression in mPFC under the indicated treatments. Immunohistochemistry of c-Fos was performed with mPFC slices as described in Section 4. The slices were re-probed with anti-DAPI antibody. Immunofluorescence images for c-Fos (green) and DAPI (blue) were acquired by confocal microscopy. (**B**) Quantitative analysis for c-Fos expression in mPFC with various treatments. Protein expression is expressed as a percentage of c-Fos fluorescence measured in the control group. Bars represent the mean ± SEM (*n* = 4). * *p* < 0.05, ** *p* < 0.01 compared with the control or CUMS group by one-way ANOVA followed by post hoc tests, respectively. (**C**) Representative immunoblots of the synaptic proteins BDNF, PSD-95, GluA1, and vGluT1 with various treatments. (**D**–**G**) Quantification of the expression of the synaptic proteins BDNF (**D**), PSD-95 (**E**), GluA1 (**F**), and vGluT1 (**G**). The expression level is expressed as a percentage of the integrated density obtained from the control group. Bars represent the mean ± SEM (*n* = 4). * *p* < 0.05, ** *p* < 0.01 by one-way ANOVA followed by post hoc tests.

We examined SYR’s effects on the expression of c-Fos, a marker of neuronal activity [38], and several synaptic proteins critical for spine synapse formation [37] in both the mPFC and hippocampus of CUMS mice. As expected, administration of fluoxetine reversed the CUMS-induced decrease in the expression of c-Fos in the mPFC in our immunohistochemistry staining assay (Figure 7A,B). By comparison, the expression of c-Fos in the mPFC of CMUS mice was also significantly elevated by the administration of SYR at the same dosage used for fluoxetine. In addition, CUMS exposure led to a reduction in the expression of several synaptic proteins in mPFC, such as BDNF, PSD-95, GluA1, and vGluT1, which could be attenuated by SYR administration (Figure 7C–G).

**Figure 8:**
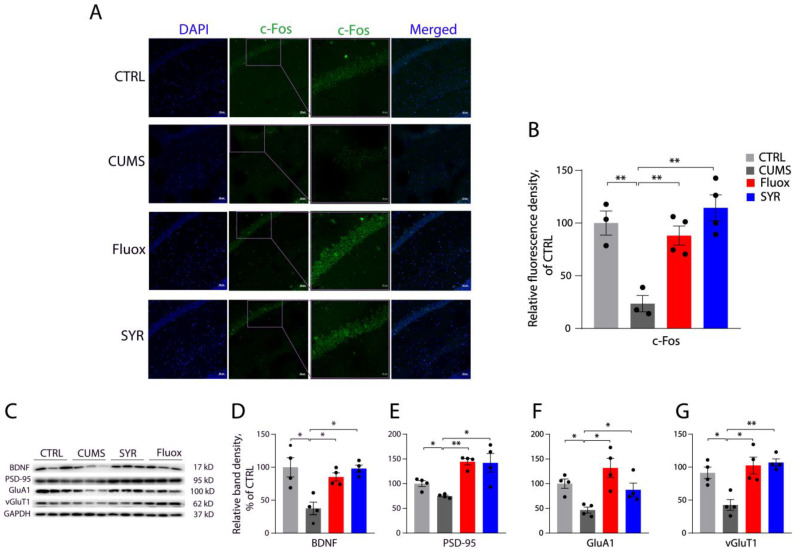
SYR effects on the expression of c-Fos and synaptic proteins in the hippocampus. (**A**) Representative immunohistochemical images of c-Fos expression in the hippocampus with the indicated treatments. (**B**) Quantitative analysis for hippocampal c-Fos expression with various treatments. Protein expression level is expressed as a percentage of c-Fos fluorescence measured in the control group. Bars represent the mean ± SEM (*n* = 4). ** *p* < 0.01 compared with the control or CUMS group by one-way ANOVA followed by post hoc tests. (**C**) Representative immunoblots of the synaptic proteins BDNF, PSD-95, GluA1, and vGluT1 with various treatments. (**D**–**G**) Quantification of the expression of synaptic proteins BDNF (**D**), PSD-95 (**E**), GluA1 (**F**), and vGluT1 (**G**). The expression level is expressed as a percentage of the integrated density obtained from the control group. Bars represent the mean ± SEM (*n* = 4). * *p* < 0.05, ** *p* < 0.01 by one-way ANOVA followed by post hoc tests.

We also investigated the synaptic effect of SYR administration in the hippocampus of mouse models (Figure 8). At the same dosage, SYR administration was shown to effectively alleviate the CUMS-induced decrease in the expression of c-Fos (Figure 8A,B) and synaptic proteins (Figure 8C–G) in the hippocampus, as fluoxetine did. Taken together, our data indicate that SYR produces a comparable antidepressant-like effect on synaptic proteins to the SSRI antidepressant fluoxetine by stimulating neuronal activity and synapse formation in both the mPFC and hippocampus in CUMS mice.

## 3. Discussion

The present study characterized one natural lignan compound, SYR, which exhibits an antidepressant-like activity in depressive animal models with a novel underlying mechanism of action by which the molecule noncompetitively inhibits SERT transport activity. All the biochemical results were supported by our molecular docking and site-directed mutagenesis analysis for the association of SYR with the S2 site in SERT. Therefore, the experimental evidence presented here demonstrated that SYR binds directly to the allosteric S2 site in SERT, and thus, noncompetitively inhibits SERT activity by blocking the conformational conversion essential for substrate transport. In addition, our data also demonstrated that the administration of SYR produces an antidepressant-like effect on animal behaviors and synaptic proteins in both the mPFC and hippocampus with a comparable efficacy to that of the conventional SSRI antidepressant fluoxetine. 

The first-line medications for depression treatment are the SSRI antidepressants. So far, all characterized SSRI antidepressants have been indicated to bind to the S1 site, and thus, to competitively inhibit 5-HT transport [23]. An allosteric site (S2) has been recently reported to be formed at the extracellular vestibule in SERT. It was proposed that the steric hindrance formed by the S2 site in the extracellular permeation pathway leads to the allosteric inhibition of substrate or ligand binding in the S1 site [27]. Compared with the S1 inhibitors, an allosteric inhibitor may exhibit a more beneficial therapeutic profile [26]. Indeed, vilazodone, an antidepressant drug approved as an allosteric inhibitor of SERT, has been clinically observed to reduce weight gain and also to be not associated with sexual dysfunction [39,40], which are the most common adverse effects of the conventional SSRI treatments. However, vilazodone also acts as a 5-HT_1A_ receptor partial agonist with a higher potency than its inhibition of SERT activity [41]. While its action on the 5-HT_1A_ receptor is assumed to provide for a faster onset of action relative to the conventional SSRIs, it is still unclear whether the dual actions translate into specific advantages in terms of treatment efficacy [42,43]. In addition, the other two allosteric inhibitors with high inhibition potency (30 nM for Lu AF60097 and 2.1 nM for Lu AF88273) have been recently developed based on the S-citalopram scaffold [27,44]. Although Lu AF60097 was demonstrated to synergistically inhibit SERT with a conventional SSRI imipramine, its limited brain availability was observed in an in vivo study [27]. On the other hand, Lu AF88273 and its homologs were shown to be brain-penetrant and subsequently to inhibit SERT expressed in rat brain synaptosomes [45]. However, clinical implication of these promising allosteric inhibitors has not been reported yet. Nevertheless, these studies have recently shifted our efforts to discover new agents targeting the S2 site in SERT. Such therapeutics using allosteric inhibitors have been proposed to potentially have fewer adverse effects because the allosteric site (S2) is much less conserved than the orthosteric site (S1) among monoamine transporters, which renders higher selectivity of the allosteric inhibitors targeting the S2 site in SERT [44]. 

Although the inhibitory potency of SYR is dozens-fold lower than those of the conventional SSRIs and allosteric inhibitors, it exerts an antidepressant-like activity comparable to the conventional SSRI inhibitor fluoxetine in our CUMS mouse models. This is possibly due to SYR’s multiple pharmacological properties, such as anti-inflammatory, antioxidant, and antidepressant actions. Inflammation and oxidative stress have been demonstrated to be the primary risk factors in the pathophysiology of depression [46]. Therefore, its multiple actions toward different targets across various biological systems may elevate its antidepressant activity and consequently display a variety of health benefits. Further investigation and validation of the antidepressant-like effects and treatment efficacy of SYR are required using more physiologically relevant models or systems, such as serotonergic neurons or brain synaptosomes, and other models, such as gendered animal models and genetically diverse models to understand its inter-individual variability and clinical translatability.

Our finding of SYR and its glycoside derivatives, as a structural subfamily of lignan compounds that exert an antidepressant activity by targeting the S2 site in SERT, opens the possibility of developing novel antidepressant agents based on SYR as a lead compound. Noticeably, SYR possesses a symmetric structure with multiple active groups, including one hydroxyl group and two dimethoxy groups at each side of the molecule. In addition, our molecular docking also indicated that SYR interacts with the side chains of various residues, such as polar, non-polar, hydrophobic, acidic, basic, and aromatic, in the allosteric S2 site of SERT. The elucidation of molecular interactions in this study would assist us in designing novel molecules with distinct pharmacodynamic profiles and specificities. It is noteworthy to emphasize that the S2 site is much less conserved among the monoamine transporters compared with the S1 site, providing possibilities for the development of more selective allosteric inhibitors toward the S2 site in SERT. Hence, it is highly expected that a further increase in inhibitory potency and specificity for the S2 site in SERT by structural modification could facilitate the development of a novel class of antidepressant drugs.

Preclinical and clinical studies have indicated that more than a dozen herbal extracts or constituents exhibit a variety of antidepressant effects in depressive animal models and patients; however, our understanding of their molecular mechanisms of action is sparse [47]. On the other hand, enormous progress in the study of the neurobiology of depression has revealed many pathophysiological factors across various biological systems and subsequently has provided many pharmacological targets that can be utilized to elucidate the mechanisms of antidepressant action of herbal constituents at the molecular level [48]. Therefore, it is feasible to demonstrate the specific interactions between herbal molecules with their targeting proteins by using the molecular pharmacology approach. The present study, together with our previous works on lignan glycoside derivatives [28,29], have provided an excellent paradigm to explore the molecular basis for the mechanism of action of those medicinal herbs with high lignan ingredient contents, such as *A. julibrissin*, *Eucommia ulmoides* Oliv., or *Eleutherococcus senticosus* Maxim., that have been commonly used as antidepressant or anxiolytic drugs in clinical practice [49,50,51].

## 4. Materials and Methods

### 4.1. Materials

SYR was purchased from Chengdu HerbSubstance Biotechnology Co. Ltd. (Batch no. PCS2285, purity: > 98%, Chengdu, China). HeLa cells (CCL-2) were from American Type Culture Collection (Manassas, VI, USA). Expression plasmids for SERT and its mutants (Y107C/C109A and S277C/X5C) in pcDNA3.1 were from the Dr. Rudnick lab (Yale University School of Medicine, New Haven, CT, USA). APP^+^, ASP^+^, protease inhibitor mixture cocktail, anti-Flag monoclonal M2 antibody, and fluoxetine were purchased from Sigma-Aldrich (Saint Louis, MI, USA). EZ-Link NHS-SS-biotin, streptavidin-agarose, Super Signal West Pico PLUS substrate, lipofectamine 2000, and Micro BCA protein assay kit were obtained from ThermoFisher Scientific (Waltham, MA, USA). MTSEA and MTSET were purchased from Biotium (Silicon Valley, CA, USA). All other reagents were of analytical grade.

### 4.2. Expression of hSERT

HeLa cells were cultured in Dulbecco’s Modified Eagle’s Medium supplemented with 10% fetal bovine serum, 100 units/mL penicillin, and 100 μg/mL streptomycin at 37 °C in a humidified 5% CO_2_ incubator. Cells grown at ~70% confluence in a 6 cm culture dish were transfected with hSERT cDNA in pcDNA3.1 by lipofectamine 2000. Transfected cells were incubated for 12 h at 37 °C with 5% CO_2_ and then transferred into 96-well plates. After being grown for an additional 12–16 h, the cells were then assayed for APP^+^ uptake or ASP^+^ binding.

### 4.3. APP^+^ Uptake and ASP^+^ Binding Assay

Measurement of transport or ligand binding activity of SERT was performed by using its fluorescent substrate (APP^+^) or ligand (ASP^+^), respectively. The cells transiently expressing WT or mutants of hSERT were applied for various treatments. Briefly, the cells were washed twice with 100 μL KRH buffer containing 20 mM HEPES (Neofroxx, Einhausen, Germany), pH 7.4, 120 mM NaCl, 1.3 mM KCl, 2.2 mM CaCl_2_, 1.2 mM MgSO_4_, and 0.1% (*w*/*v*) glucose. APP^+^ uptake was measured by adding 100 μL KRH buffer containing 2 μM APP^+^ and incubating for 5 min at 22 °C. Excess APP^+^ was then removed by 3× rapid washing with 100 μL KRH buffer each time. The extent of APP^+^ accumulated in the cells was determined by fluorescence spectrometry with an Infinite 200 Pro microplate reader (Tecan, Männedorf, Switzerland). 

ASP^+^ binding to SERT expressed on the cell surface was measured with digitonin-permeabilized cells as described previously [29]. The cells expressing WT or mutants of hSERT were incubated with 10 μM ASP^+^ in the presence of 25 μg/mL digitonin at 22 °C for 5 min. After removing excess ASP^+^ by 3× rapid washing, ASP^+^ fluorescence retained in the cell membrane was determined by fluorescence spectrometry. Nonspecific APP^+^ transport or ASP^+^ binding was measured in the presence of 100 μM fluoxetine and subtracted to give APP^+^ uptake or ASP^+^ binding, respectively.

### 4.4. Cystine Accessibility Measurements

We employed a cysteine-modification approach to determine conformational states of SERT with fluorescent ligands by measuring accessibility of the reactive cysteine residues placed in either the extracellular or cytoplasmic pathway to MTS reagents, as described previously [33]. The cysteine mutant, Y107C/C109A or S277C/X5C, was used for examining conformational changes in the extracellular or cytoplasmic pathway, respectively. For accessibility measurement in the extracellular pathway, reactivity of Y107C/C109A with MTSET, a membrane impermeant cysteine reagent, was determined by examining inhibition of APP^+^ uptake of the cysteine mutant Y107C/C109A by MTSET over a range of concentrations [33]. The IC_50_ for MTSET inhibition was then determined and converted to the rate constant of Y107C/C109A reactivity with MTSET, as described previously [52].

Similarly, we used S277C/X5C as an indicator to measure conformational changes in the cytoplasmic pathway by using digitonin-permeabilized cells, as described previously [33]. The IC_50_ for MTSEA inhibition was determined and converted to the rate constant of S277C/X5C with MTSEA.

### 4.5. Biotinylation and Immunocytochemistry

Cell surface expression of C-terminal Flag-tagged hSERT was determined using a membrane-impermeant biotinylation reagent sulfo-NHS-SS-biotin, as described previously [53]. In brief, HeLa cells expressing WT-hSERT were incubated twice with sulfo-NHS-SS-biotin for 20 min on ice. After labeling, the cells were rinsed with 100 mM glycine in phosphate-buffered saline (PBS) buffer (Biosharp, Beijing, China) for 20 min to quench excess reagent. The cells were then lysed, and biotinylated proteins were recovered using streptavidin-agarose beads in an overnight incubation at 4 °C with gentle agitation. The beads were washed, and biotinylated proteins were eluted with 50 μL of SDS-PAGE sample buffer. The eluates were applied to a 10% SDS-polyacrylamide gel, and hSERT was detected by immunoblotting with anti-Flag monoclonal M2 antibody (1:1000). Immunoreactive bands were visualized by chemiluminescence, and the amount of cell surface expression of SERT was quantified using a UVP Biochemi II imaging system (Upland, CA, USA).

The immunocytochemistry protocol was adapted from the previous work [54]. Briefly, the cells expressing WT-hSERT seeded onto poly-*D*-lysine-coated glass slips in a 12-well plate were incubated with KRH buffer containing 0.5 μM SYR or 10 μM fluoxetine for 5 min at 22 °C, respectively, followed by fixation with 4% paraformaldehyde for 15 min. The cells were then permeabilized with 0.1% Triton X-100 for 7 min on ice and incubated with anti-Flag M2 antibody overnight at 4 °C with gentle shaking, followed by treatment with the secondary antibody conjugated to fluorophore 594 (1:100) for 1 h at 22 °C. Immunofluorescence images were captured at 60× using the Zeiss LSM 900 confocal microscope (Oberkochen, Germany) and then analyzed using Zen Blue software (blue edition v3.1). Intracellular fluorescence intensity was subtracted from total fluorescence in each cell to obtain fluorescence intensity of hSERT in the cell plasma membrane. 

### 4.6. Molecular Docking

Molecular docking was conducted by using Glide module in Schrödinger Suites v2021.2 on the structure of hSERT (PDB ID, 7LWD, 3.65 Å) [26]. The template structure of SERT was optimized for hydrogen bonding network, conformation of bonds, and energy constraints using the Protein Preparation Wizard, while optimization of the SYR molecule for its conformation and energy was carried out in the OPLS4 force field. Molecular docking was conducted under a standard precision, which generated 20 poses of SYR in SERT with van der Waals radius scaling of 0.8 for the protein and ligand. After conformational search and energy minimization, one pose with the highest glide score was exported into PyMOL v2.5.2 for visualization.

### 4.7. Site-Directed Mutagenesis

hSERT mutants were generated using the Mut Express II Fast Mutagenesis kit (Vazyme, Nanjing, China). Briefly, forward and reverse primers were used to amplify a full-length hSERT. The PCR products were then digested with restriction enzymes, excised from agarose gels, and ligated into pcDNA3.1. All mutations were confirmed by full-length DNA sequencing.

### 4.8. Animal Handling and Drug Administration

C57BL/6 adult (6–8 weeks, body weight, 18–22 g) male mice were maintained and handled in a standard condition at the Experimental Animal Center of Southern Medical University (Guangzhou, China). Animal use was approved by the Animal Use and Care Committee of Southern Medical University (approved code: SMUL2021177, approved date: 12 December 2021).

The CUMS procedure was carried out according to a protocol described previously [55]. Except for those in the control group, mice were randomly exposed to two mild unpredictable stressors per day for 3 weeks. The stressors included water deprivation, restraint, tilted cage, wet/no bedding, rotation, peppermint, noise, and inverted light off/on. Mice in the control group were group housed without any stressors throughout the procedure. Under our CUMS procedure, a very small number of mice did exhibit resilience phenotypes, which is very common but also a limitation of the study using CUMS animal models. 

Saline, fluoxetine, or SYR diluted with 30% PEG400 was administered by intraperitoneal injection (i.p.) with a dose of 10 mg/kg for fluoxetine or 5, 10, or 20 mg/kg for SYR once per day, respectively. Mice were continuously administered with fluoxetine or SYR from day 7 to day 21 during the CUMS procedure. According to experimental requirements, mice were divided into 6 groups (10 mice per group): control, CUMS, fluoxetine, SYR-L (low dosage, 5 mg/kg), SYR-M (middle dosage, 10 mg/kg), and SYR-H (high dosage, 20 mg/kg), respectively.

### 4.9. Behavioral Tests

The animal procedure for behavioral tests is shown in Figure 6A. NSFT was performed after 16 h food deprivation, as described previously [56]. The mouse was placed in an open field (24 × 40 × 14 cm), where a new chow was put in the center, and the latency time to bite food was counted over 10 min. Home cage feeding within a 20 min period was measured after the test. FST was carried out according to a protocol described previously [57]. The mouse was placed into a beaker (Bomex, Beijing, China) with a height of 18 cm filled with water (22 ± 1 °C) for 6 min. The immobile time was measured during minutes 2–6. TST was performed according to a previous description [58]. The mouse was suspended from a hook that hung from a suspension bar by placing a piece of adhesive tape on its tail about 1 cm away from the tip. The immobile time during minutes 2–6 was measured. 

### 4.10. Immunohistochemistry and Immunoblot Analysis

Mouse mPFC and hippocampal slices were prepared as previously described [37]. Briefly, mice were deeply anesthetized with 4% pentobarbital sodium (Sigma-Aldrich, Saint Louis, MI, USA), and perfused transcardially with PBS, followed by 4% phosphate-buffered paraformaldehyde. The brains were then cut into serial 3–4 μm thick coronal sections that were then incubated with anti-c-Fos primary antibody (1:100, Abcam, Cambridge, UK) in blocking solution (5% normal goat serum and 0.2% Triton X-100 in PBS) overnight at 4 °C, followed by incubating with anti-rabbit IgG Fluor 488 (1:500, Cell Signaling, Danvers, MA, USA) for an additional 1 h at 22 °C. All slices were then re-probed with DAPI (Solarbio, Beijing, China), and immunofluorescence images for c-Fos and DAPI were acquired by confocal microscopy with the Zeiss LSM 900 confocal microscope (Oberkochen, Germany). 

Synaptic proteins were extracted from mPFC or hippocampus tissues using RIPA lysis buffer, respectively. Expression of the indicated proteins under various treatments was then analyzed by immunoblotting, as previously reported [37]. Primary antibodies used in our experiments include rabbit anti-PSD-95 (1:1000, Cell Signaling, Danvers, MA, USA), rabbit anti-GluA1 (1:1000, Cell Signaling, Danvers, MA, USA), rabbit anti-vGLUT1 (1:1000, Cell Signaling, Danvers, MA, USA), and rabbit anti-GAPDH (1:1000, Cell Signaling, Danvers, MA, USA). Secondary antibodies were applied at 1:1000 dilution. Quantification of immunoreactive protein bands was performed using Image J v1.53u, and GAPDH was used as an internal reference to normalize expression levels of those proteins, respectively.

### 4.11. Data Analysis

Nonlinear regression fits of experimental and calculated data were performed with Origin (Origin Lab, Northampton, MA, USA). The statistical analysis given was from multiple experiments. Data with error bars represent the mean ± standard error of mean (SEM) for at least three experiments. Statistical analysis was performed using one-way ANOVA followed by post hoc tests, and statistical significance was set at *p* < 0.05.

## 5. Conclusions

The present study characterized the pharmacological profile of an herbal molecule SYR in inhibiting SERT by using biochemical, pharmacological, and behavioral approaches. Our results indicated that SYR noncompetitively inhibits SERT activity by stabilizing SERT in an outward-open and inward-closed conformation. In addition, our molecular docking and site-directed mutagenesis demonstrated that SYR binds to the allosteric site in SERT. Moreover, SYR administration was shown to reverse CUMS-induced abnormalities in the behaviors and expression of synaptic proteins in depressive animal models. The study not only provides new insights into the molecular basis for *A. julibrissin* in the treatment of depression, but also opens the possibility to develop a new class of the allosteric site-targeted antidepressants with a novel mechanism of action.

## Figures and Tables

**Table 1:** Transport and binding kinetics for SERT WT and mutants.

hSERT	Mutating Site	K_m_ for APP^+^ (μM)	V_max_ (AFU/min)	SYR IC_50_ for Transport (μM)	SYR IC_50_ for Binding (μM)
WT		2.24 ± 0.45	22.98 ± 0.92	0.25 ± 0.01	0.96 ± 0.02
Q111N		3.69 ± 0.36	26.93 ± 1.35	17.72 ± 0.45 ***	114.96 ± 2.47 ***
I327A		4.27 ± 0.79	26.54 ± 1.43	19.53 ± 1.02 ***	53.22 ± 7.01 ***
D328A		3.81 ± 0.27	27.83 ± 0.85	19.59 ± 1.21 ***	39.21 ± 0.78 ***
E493N		4.83 ± 0.19 *	25.48 ± 0.96	9.16 ± 0.34 ***	13.47 ± 1.30 **
E494Q		6.83 ± 0.86 *	31.12 ± 0.89	11.97 ± 2.17 ***	9.77 ± 0.78 **
T497A		3.85 ± 0.06	26.63 ± 1.09	10.60 ± 2.40 ***	43.74 ± 2.60 ***
G498T		3.75 ± 0.32	26.30 ± 1.52	40.46 ± 0.54 ***	91.01 ± 6.29 ***
P499G		3.72 ± 0.45	27.28 ± 0.43	11.01 ± 1.43 **	46.83 ± 2.35 ***
F556A	S2	4.69 ± 1.02	26.84 ± 1.74	7.39 ± 1.12 **	21.63 ± 1.20 ***
S559A		3.82 ± 0.55	23.68 ± 0.57	19.51 ± 1.46 ***	36.84 ± 2.49 ***
P560G		1.85 ± 0.16	21.70 ± 0.90	5.99 ± 0.96 **	14.67 ± 0.26 **
P561G		2.73 ± 0.09	26.55 ± 1.08	46.31 ± 0.94 ***	130.75 ± 5.72 ***
Q562N		1.85 ± 0.36	23.57 ± 1.49	48.78 ± 4.51 ***	99.95 ± 2.04 ***
L563A		1.53 ± 0.07	23.53 ± 0.45	6.28 ± 0.57 **	39.01 ± 0.92 ***
R564A		3.41 ± 0.12	27.10 ± 0.08	14.60 ± 1.59 ***	30.45 ± 1.42 ***
L565A		0.68 ± 0.04 *	21.13 ± 0.16	9.98 ± 1.22 ***	20.96 ± 0.45 ***
Y579A		2.81 ± 0.08	23.89 ± 2.33	16.30 ± 1.06 ***	28.62 ± 0.15 ***
T583A		3.68 ± 0.15	26.97 ± 0.26	8.68 ± 1.40 ***	30.88 ± 0.93 ***
Y95F		ND	ND	2.64 ± 0.12 *	1.21 ± 0.01
I172F	S1	ND	ND	2.22 ± 0.04 *	1.51 ± 0.04
S438T		ND	ND	0.91 ± 0.02 *	2.32 ± 0.02

APP^+^ transport or ASP^+^ binding was performed on intact or digitonin-permeabilized cells transiently expressing WT, S2, or S1 mutants of hSERT as described in Section 4. SYR was added 5 min prior to APP^+^ or ASP^+^ to obtain equilibrium. Data are shown as the mean ± SEM from at least three experiments (*n* ≥ 3). * *p* < 0.05; ** *p* < 0.01; *** *p* < 0.001 compared with WT by one-way ANOVA followed by post hoc tests. ND, not detected.

## Data Availability

The data presented in this study are available on request from the corresponding author.

## Supplementary Materials

The following supporting information can be downloaded at: https://www.mdpi.com/article/10.3390/ph17121637/s1, Table S1: 5-HT displacement of APP^+^ uptake for SERT WT and S2 mutants; Figure S1: Average body weight changes in mice during drug administration period.
