# Supplementary material for: (-)-Syringaresinol Exerts an Antidepressant-like Activity in Mice by Noncompetitive Inhibition of the Serotonin Transporter
Source: Pharmaceuticals (Basel). 2024 Dec 5;17(12):1637. doi: 10.3390/ph17121637 (PMC11678425; doi:10.3390/ph17121637)
Supplement: Supplementary file 1 [file pharmaceuticals-17-01637-s001.zip › pharmaceuticals-3337065-supplementary.pdf]

Table S1. 5-HT displacement of APP[+] uptake for SERT WT and S2 mutants.

hSERT	5-HT K_i (μM)
WT	0.66 ± 0.09
Q111N	0.14 ± 0.01*
I327A	0.54 ± 0.08
D328A	0.61 ± 0.06
E493N	0.31 ± 0.02*
E494Q	0.72 ± 0.04
T497A	0.78 ± 0.05
G498T	0.26 ± 0.02*
P499G	0.36 ± 0.01
F556A	0.25 ± 0.01*
S559A	0.25 ± 0.02*
P560G	0.84 ± 0.05
P561G	0.30 ± 0.01*
Q562N	0.26 ± 0.04*
L563A	0.35 ± 0.02*
R564A	0.91 ± 0.03
L565A	73.89 ± 2.34***
Y579A	0.97 ± 0.17
T583A	2.37 ± 0.27**

5-HT displacement of APP^+ uptake was performed on the cells transiently expressing SERT WT or mutants by incubating APP^+ in the presence of 5-TH at a range of concentrations as described under Section 4. The K_i values for 5-HT displacement were calculated from non-linear regression analysis of APP^+ uptake. Data were shown as the mean ± SEM from at least three experiments. *$p < 0.05$; **$p < 0.01$; ***$p < 0.001$ compared with WT by one-way ANOVA.

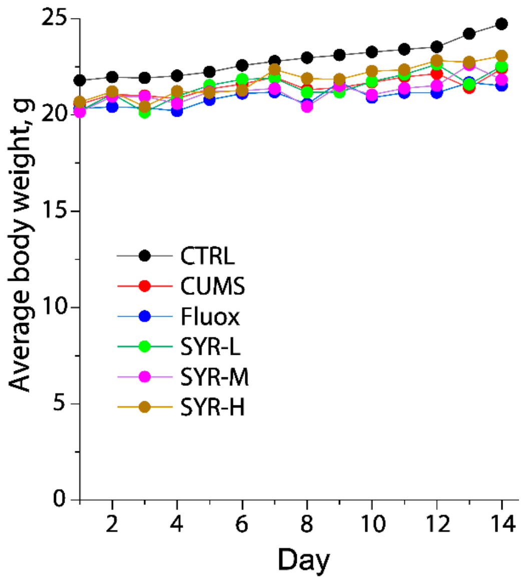

Figure S1. Average body weight changes of mice during drug administration period. The body weight of individual mouse was measured daily during 14-day drug administration period. The graph represents time-dependent changes in average body weight of mice in each group.
